# Ophthalmic manifestations in children with Down Syndrome in Bogotá, Colombia

**DOI:** 10.1186/s12886-023-02863-y

**Published:** 2023-05-15

**Authors:** William Rojas‑Carabali, María Camila Cortés-Albornoz, Gabriela Flórez‑Esparza, Carlos Cifuentes‑González, Alejandra de‑la‑Torre, Claudia Talero‑Gutiérrez

**Affiliations:** Neuroscience (NEUROS) Research Group, School of Medicine and Health Sciences, NeuroVitae Center for Neuroscience, Institute of Translational Medicine (IMT), Universidad del Rosario, Carrera 24 # 63C 69, Bogotá, Colombia

**Keywords:** Down Syndrome, Ophthalmology, Ocular manifestation, Eye, Cross-sectional

## Abstract

**Background:**

To describe the ocular features of a cohort of children with Down Syndrome (DS) in Bogotá, Colombia.

**Methods:**

We performed a cross-sectional study, evaluating 67 children with DS. A pediatric ophthalmologist performed a complete optometric and ophthalmological evaluation of each child, including visual acuity, ocular alignment, external eye examination, biomicroscopy, auto-refractometry, retinoscope in cycloplegia, and fundus examination. Results were reported as frequency distribution tables with percentages for categorical variables and means and standard deviation or median and interquartile ranges for continuous variables, according to their distribution. We used the Chi-square test or Fisher’s exact test for categorical variables and ANOVA or Kruskal–Wallis for continuous variables when indicated.

**Results:**

A total of 134 eyes from 67 children were evaluated. Males represented 50.7%. The children’s age ranged from 8–16 years, with a mean of 12.3 (SD 2.30). The most frequent refractive diagnosis per eye was hyperopia (47%), followed by myopia (32.1%) and mixed astigmatism (18.7%). The most frequent ocular manifestations were oblique fissure (89.6%), followed by amblyopia (54.5%) and lens opacity (39.4%). Female sex was associated with strabismus (*P* = 0.009) and amblyopia (*P* = 0.048).

**Conclusion:**

Our cohort had a high prevalence of disregarded ophthalmological manifestations. Some of these manifestations, such as amblyopia, can be irreversible and severely affect the neurodevelopment of DS children. Therefore, ophthalmologists and optometrists should be aware of the visual and ocular affection of children with DS to assess and provide appropriate management. This awareness could improve rehabilitation outcomes for these children.

## Key messages

- Knowing the most frequent ocular manifestations of children and adolescents with Down syndrome is relevant to identify them early.
- Early identification of ophthalmological manifestations helps prevent neurodevelopmental disorders such as amblyopia.
- There are differences in neglected ophthalmological manifestations such as amblyopia between boys and girls.

## Background

Down syndrome (DS) is the most common chromosomal anomaly with a vast constellation of characteristic findings. It is frequently associated with intellectual disability, delays in physical growth, distinctive facial features, hands with a single palmar crease, and congenital heart defects. In like manner, individuals with DS have a higher incidence of functional and structural abnormalities in the eyes [1], with a prevalence of ocular manifestations in up to 85% of the children with DS [2]. Numerous studies have reported abnormalities of the eyelid, lacrimal drainage, cornea, iris, lens, retina, and optic disc, as well as ametropia, amblyopia, strabismus, and nystagmus in children with DS. However, all reports have no consistent incidence patterns of each ocular defect.

Furthermore, most studies reporting ophthalmic features in DS have been performed in the Caucasian and some in other Asian and African populations [3–7]. However, there is scarce literature on ocular alterations in Latin American pediatric patients with DS [8]. Therefore, our study aimed to describe the ocular features of a cohort of children with DS in Bogotá, Colombia.

## Methods

### Study design

We performed a cross-sectional study following the STROBE guidelines to report our results.

### Setting and participants

We evaluated noninstitutionalized children with DS who attended “Corporación Síndrome de Down” in Bogotá, Colombia, from October 2021 to June 2022.

We used a non-probabilistic sampling by convenience, including children under 18 years old with a DS diagnosis confirmed with a genetic test (including trisomy 21, translocation, or mosaicism) whose parents accepted participation and signed informed consent. A total of 67 children and adolescents (33 females and 34 males). A list of all children attending “Corporación Síndrome de Down” was obtained, totaling 188 children. Participants were invited to participate voluntarily via phone call. The first 67 children who answered the call were evaluated.

### Procedures

Written informed consent was obtained from the children’s parents or legal representatives. A pediatric ophthalmologist performed a complete optometric and ophthalmological evaluation of each child, including visual acuity (VA), tested using the conventional Snellen chart, the illiterate E chart, and Lea picture charts or Lea paddles (preferential looking) test depending on their abilities. Ocular alignment was evaluated using the prism cover test, and the near point of convergence (NPC) was assessed using a fixation target brought in towards the child’s eye from 40 cm, with the distance where one eye begins to deviate measured from the respondent’s lateral canthus with a tape measure. Then, a detailed external eye examination and biomicroscopy evaluation with the slit lamp was performed. The auto KR-800 kerato-refractometer (Topcon Corporation, Tokyo, Japan) was used to objectively determine the refractive error and the keratometry (K). Corneal curvatures with K2 > 47.2 were considered keratoconus suspect. Additionally, the refractive status was confirmed using the retinoscope in cycloplegia with 1% cyclopentolate. Due to the lack of cooperation, it was impossible to perform cycloplegic refractometry on all the DS participants; in those cases, auto-refractometry was reported. Refractive errors were defined considering previous literature [9–11] as follows: myopia as Spherical Equivalent Refraction (SER) of − 0.50 D or less, emmetropia as SER greater than − 0.50 D and less than + 2.00 D, hyperopia as SER of + 2.00 D or greater, high hyperopia as SER > 5.00 D, and high myopia as SER of − 6.00 D or greater [12]. SER was calculated as follows: sphere + 0.5*cylinder. Additionally, considering that the previously described classification could misclassify as emmetropes, most children with high mixed astigmatism, we classified refractive errors using a multicomponent approach to better demonstrate the prevalence of refractive errors. For this, we used the calculator and classification provided by Galvis et al. [13]. Anisometropia was considered when there was an interocular difference in the SER of 1 D or more [14]. Additionally, dilated fundus examination was performed using binocular indirect ophthalmoscopy focusing on the optic nerve head, macula, and periphery.

### Statistical analysis

Univariate analysis was done on all variables. The results were reported as frequency distribution tables with percentages for categorical variables and means and standard deviation or median and interquartile ranges for continuous variables, according to their distribution. Shapiro–Wilk test was used to probe normal distribution in each variable. To evaluate associations between categorical variables, we used the Chi-square independence test and Fisher exact test when indicated. ANOVA and Kruskal–Wallis were used for continuous variables when indicated. A *p*-value ≤ 0.05 was considered statistically significant. All data were analyzed using the Jamovi (Version 1.6).

### Ethical considerations

This study was approved by the Ethics Committee of the Universidad del Rosario under the reference CVO 005 717-CV1069 and conducted according to the tenets of the Helsinki Declaration.

## Results

We evaluated 134 eyes from 67 children, of which males represented 50.7% and 53.8% proceeded from low-income families. The children’s age ranged from 8 to 16 years, with a mean of 12.4 (SD 2.28) years. The mean age at birth was 36.6 weeks (SD 2.96 weeks), the mean birth weight was 2,394 g (SD 710 g), and 89.1% required management in the neonatal intensive care unit after birth. Regarding the use of glasses, 80.3% of children used them, and the mean age of onset of glasses use was 5.1 years (SD 3.2 years). Female sex was statistically associated with the use of glasses (*P* = 0.03); moreover, females tended to use glasses earlier than males (Median 3.5 vs. 5 years, respectively). Additionally, three patients had history of strabismus surgery, two of lacrimal syringing, and one of lens extraction. Information about the socio-demographic characteristics is available in Table 1.

**Table 1:** Socio-demographic characteristics

**Characteristic**	**Result**
**Age (mean, SD)**	12.4 ± 2.28 years
**Sex (n, %)**	Male = 34 (50.7%) Female = 33 (49.3%)
**Age at birth (mean, SD)**	36.6 ± 2.96 weeks
**Socio-economic stratification** ^a^	1 = 9.0% 2 = 44.8% 3 = 34.3% 4 = 10.4% 5 = 0% 6 = 1.5%
**Health coverage**	Public Healthcare Plan = 20.9% Private Healthcare Plan = 79.1%

^a^ It is a Colombian socio-economic index derived from the income levels (low income: 1 and2; intermediate income 3 and 4; high income: 5 and 6

### Refractive errors

**Fig. 1:**
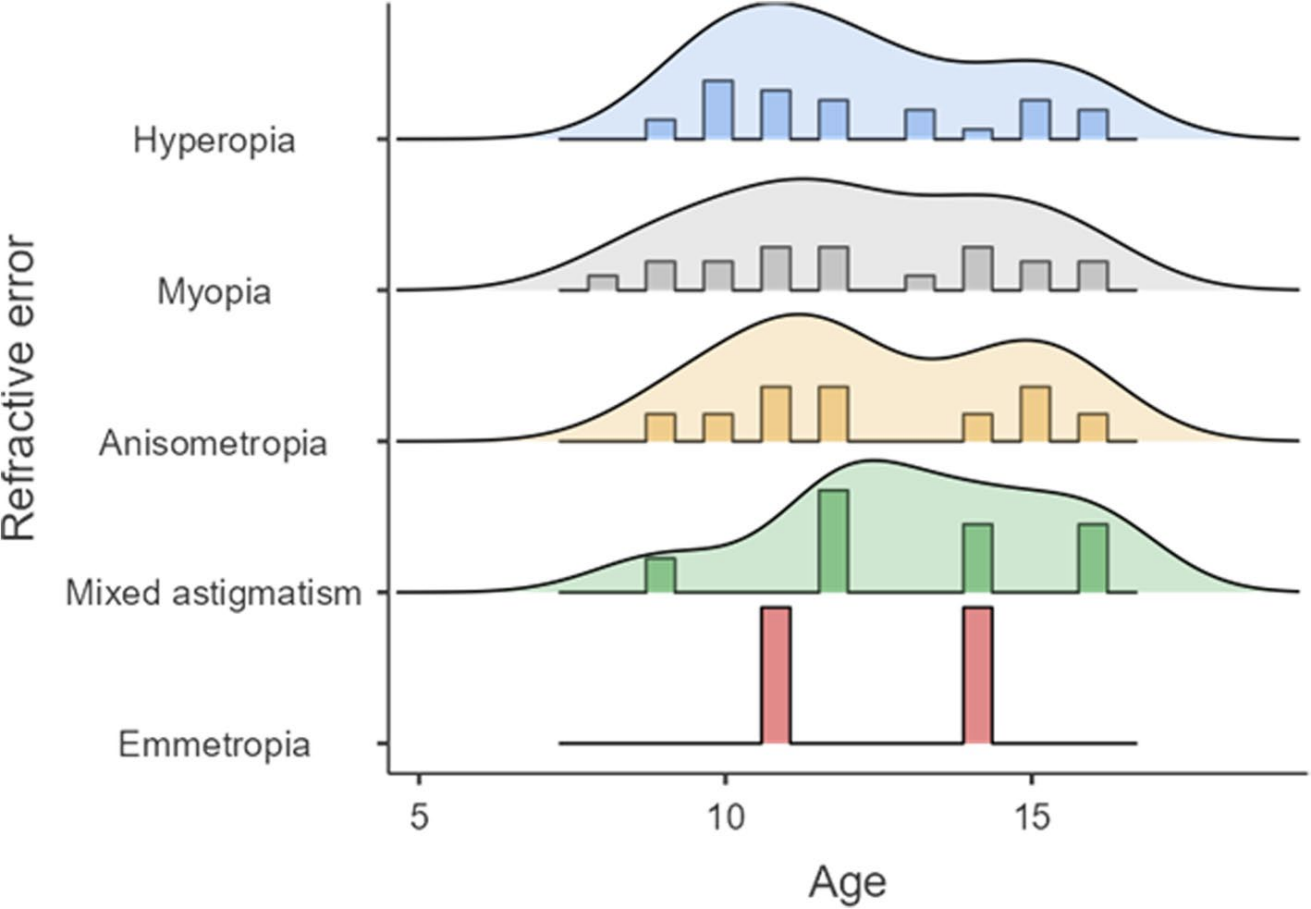
Distribution of refractive errors by age

The refractive errors were analyzed by the classification based on the SER (Table 2) and by a multiple components’ classification at eye level (Table 3) and subject level (Table 4 and Fig. 1). As for the SER classification, it was found that the most frequent refractive error was hyperopia which was identified at 23.9%, followed by myopia at 20.9%, and high myopia at 14.9%. As expected, all the components of the refractive indexes, except the cylinder, were statistically different between the refractive error groups (SER [*p* =  < 0.001], sphere [*p* =  < 0.001], and cylinder [*p* = 0.271]). Additionally, these differences between refractive error groups were also evidenced in best-corrected visual acuity (BCVA) and uncorrected visual acuity (UCVA) (BCVA *p* = 0.009; UCVA *p* = 0.05). For more detailed information on each refractive index and VA according to the SER classification, see Table 2.

**Table 2:** Refractive error classified by spherical equivalent

**Refractive Error**	Eyes *n* = 134 (%)	**Refractive Indexes**			**Visual Acuity**
		Spherical equivalent Mean ± SD (D)	Sphere Mean ± SD (D)	Cylinder Mean ± SD (D)	BCVA Mean ± SD (LogMAR)
					UCVA Mean ± SD (LogMAR)
**Myopia**	28 (20.9)	-2.74 ± 1.50	-1.21 ± 1.46	-3.07 ± 1.32	0.57 ± 0.33
					0.76 ± 0.39
**High Myopia**	20 (14.9)	-12–88 ± 3.86	-11.5 ± 4.02	-2.78 ± 1.70	0.78 ± 0.43
					0.94 ± 0.49
**Hyperopia**	32 (23.9)	3.42 ± 0.87	4.84 ± 1.28	-2.84 ± 1.81	0.47 ± 0.26
					0.75 ± 0.29
**High Hyperopia**	9 (6.7)	5.26 ± 0.53	6.58 ± 1.13	-2.64 ± 1.76	0.34 ± 0.15
					0.50 ± 0.39
**Emmetropia**	45 (33.6)	0.91 ± 0.66	2.08 ± 1.05	-2.34 ± 1.48	0.44 ± 0.23
					0.56 ± 0.29

Anisometropia was found in 10 (14.9%) children

*BCVA* Best-Corrected Visual Acuity, *D* Diopters, *SD* Standard deviation, *UCVA* Uncorrected Visual Acuity

ANOVA (Kruskal–Wallis) shows a statistically significant difference in VA and all refractive indexes except in Cylinder between the groups with a *p* < 0.05

**Table 3:** Refractive error at eye-level classified by the multicomponent definitions [10]

**Refractive error**	Eyes *n* = 134 (%)	**Refractive Indexes**			**Visual Acuity**
		Spherical equivalent Mean ± SD (D)	Sphere Mean ± SD (D)	Cylinder Mean ± SD (D)	BCVA Mean ± SD (LogMAR)
					UCVA Mean ± SD (LogMAR)
**Hyperopia**	63 (47%)	2.90 ± 1.55	4.05 ± 1.94	-2.29 ± 1.45	0.42 ± 0.24
					0.64 ± 0.32
**Myopia**	43 (32.1%)	-7.67 ± 5.64	-6.11 ± 5.75	-2.92 ± 1.55	0.72 ± 0.40
					0.83 ± 0.45
**Mixed Astigmatism**	25 (18.7%)	0.41 ± 0.98	2.22 ± 1.53	-3.61 ± 1.54	0.49 ± 0.23
					0.64 ± 0.28
**Emmetropia**	3 (2.2%)	-0.04 ± 0.38	0.25 ± 0.50	-0.58 ± 0.29	nd
					0.47 ± 0.46

*BCVA* Best-Corrected Visual Acuity, *D* Diopters, *nd* No data, *SD* Standard deviation, *UCVA* Uncorrected Visual Acuity

ANOVA (Kruskal–Wallis) shows a statistically significant difference between the groups with a *p* < 0.05 in all refractive indexes and BCVA

**Table 4:** Refractive error at subject-level classified by the multicomponent definitions [10]

**Classification**	**n children (%)**	**7 to 9 years (%)**	**10 to 12 years (%)**	**13 to 15 years (%)**	**16 to 17 years (%)**
**Hyperopia**	28 (41.8)	2 (28.5)	15 (46.9)	8 (40)	**3 (37.5)**
**Myopia**	20 (29.9)	3 (42.8)	8 (25)	6 (30)	2 (25)
**Anisometropia**	10 (14.9)	1 (14.2)	5 (15.6)	3 (15)	1 (12.5)
**Mixed astigmatism**^a^	8 (11.9)﻿	1 (14.2)	3 (9.3)	2 (10)	2 (25)
**Emmetropia**	1 (1.5)	0 (0)	1 (3.1)	1 (5)	0 (0)
**Total**^b^	67 (100)	7 (10.4)	32 (47.7)	20 (29.8)	8 (11.9)

^a^ 65 (97%) children had astigmatism < -0.75cyl in at least one eye

^b^ To calculate the percentages in the total raw the denominator was 67 children. In the columns, the denominators were their respective totals

Regarding the refractive defects classified by the multicomponent classification [13], the most frequent diagnosis per eye was hyperopia (47%), followed by myopia (32.1%), and mixed astigmatism (18.7%). Additionally, there were statistically differences between the refractive error groups in all the refractive indexes, including cylinder (SER [*p* =  < 0.001], sphere [*p* =  < 0.001], and cylinder [*p* =  < 0.001]), and visual acuity (BCVA *p* = 0.009; UCVA *p* = 0.085). However, it must be considered that it was impossible to obtain the VA data in some cases, so there is a “no data” in Table 3. Additionally, the reactive errors were classified at a subject level, as evidenced in Table 4 and Fig. 1.

### Corneal curvature

The K1 mean (*n* = 121 eyes) was 45.0 (SD 1.94) with a range of 42.0 to 51.3, and the K2 mean (*n* = 120 eyes) was 47.5 (SD 1.96) with a range of 43 to 56.3. For detailed information regarding the keratometry of each eye, see Table 5. Although we only used K2 values to classified patients as keratoconus suspect, these patients have statistically significant differences in all the keratometry measurements compared with those without keratoconus (K1 OD [*P* =  < 0.001]; K2 OD [*P* =  < 0.001]; K1 OS [*P* = 0.001]; K2 OS [*P* =  < 0.001]). Additionally, there were significant differences in the mean of the cylinder measurement (OD *P*: 0.05, OS *P* = 0.031) between children with and without keratoconus suspect, but not in the sphere and SER measurement. Additionally, there were significant differences in the SER between children with and without amblyopia OU (OD *P* = 0.04 and OS *P* = 0.02), but not in the SER between glasses-wearing and non-wearing children. Moreover, the difference between the SER in the real refractive formula and the glasses in use exceeded 1.5 D on average, reaching 10 D in some cases. OD differed on 1.54 ± 1.99 D with a range of 0.0—9.87 D, and OS differed 1.50 ± 1.91 D with a range of 0.0 – 10 D.

**Table 5:** Keratometry

**Parameter**	**Eyes ** ***n*** ** = 121 (%)**	**Keratometry** **Mean ± SD**	**Min**	**Max**
**K1 of all eyes**	121	45 ± 1.94	42	51.3
**K2 of all eyes**	120	47.5 ± 1.96	43	56.3
**K1 OD**	61	45.1 ± 1.95	42	51.3
**K2 OD**	61	47.6 ± 2.08	43.8	56.3
**K1 OS**	60	45 ± 1.96	42.3	50.3
**K2 OS**	59	47.5 ± 1.83	43	52.3

### Ocular manifestations

Ocular manifestations were present in 100% of the children, including ametropias. After refractive errors, the most frequent ocular manifestations were oblique fissures (89.6%), followed by amblyopia (54.5%) and lens opacity (39.4%). In the bivariate analysis, amblyopia was statistically associated with using glasses (*P* = 0.009). Likewise, the female sex was associated with strabismus (*P* = 0.009), amblyopia (*P* = 0.048), and Hirshberg misalignment (*P* = 0.021).

Nystagmus was observed in 19 children (28.4%), most frequently jerk nystagmus followed up by the horizontal-pendular type. Keratoconus was suspected in 36 (53.7%) due to their elevated flat and steep keratometry (K1 and K2), and 3 (4.4%) already had a confirmed diagnosis. Nasolacrimal duct obstruction or dacryostenosis was noted in 13 children (19.4%), and strabismus was found in 14 (21.5%), of which 12 presented esodeviations and 2 had exodeviations. On fundus examination, the most common feature was myopic choroidosis (10.4%). More detailed information regarding the ocular features is found in Table 6.

**Table 6:** Ophthalmic features found in the current study compared with the findings worldwide

**Ocular feature**	Current study Colombia 2022 *n* = 67	**South America** Da Cunha & De Castro Moreira [5] Brazil 1996 *n* = 152	**Africa** Adio & Wajuihian [4] Nigeria 2012 *n* = 42	**Western Asia** Ugurlu & Altinkurt [12] Turkey 2020 *n* = 44	**Eastern Asia** Kim et al. [3] Korea 2002 *n* = 123	**Europe** Fimiani et al. [13] Italy 2007 *n* = 157	**North America** Tsiaras et al. [14] USA 1999 *n* = 68
**Age mean** **range**	12.4 years [8–16 years]	[2 months to 18 years]	11.43 years [6 months to 28 years]	13.10 years [7 to 18 years]	6.5 years [6 months to 9 years]	5.28 years [0–18 years]	10.6 years [5–19 years]
**Refractive errors classifications**	Refractive error at subject-level classified by the multicomponent definitions [10]	Hyperopia (SE over 0.50 D) Myopia (SE under -0.50 D) Astigmatism (> 0.50 D cyl)	Hyperopia (≥ + 1.00 D) Myopia (≤ − 0.50 D) Astigmatism (≤ − 0.75 cyl)	Hyperopia (over 0.50 D) Myopia (under 0.50 D) Astigmatism (over 1 D cyl)	Emmetropia (0.75 D and + 0.75 D SE). Myopia (< 0.75 D SE), hyperopia (> + 0.75 D SE), and Astigmatism pure (> 0.75 D cyl)	Emmetropia (− 0.75 D and + 0.75 D SE) Myopia (less than − 0.75 D) Hyperopia (more than + 0.75 D) Astigmatism (more than 0.75 D of cyl) Anisometropia (difference between the two eyes of more than 1 D)	High myopia (more than -5D) High hyperopia (more than 4D) Astigmatism (more than 1.75 D of cyl)
	n children (%)						
**Emmetropia**	1 (1.5)	3 (1)	10 (23.8)	-	57 (46.3)	-	-
**Myopia**	20 (29.9)	19 (13)	16 (38.1)	13 (29.5)	31 (25.2)	14 (9)	16 (23.5)
**Hyperopia**	28 (41.8)	39 (26)	4 (9.5)	31 (70.5)	35 (28.5)	93 (59)	High hyperopia 3 (4.4)
**Mixed astigmatism**	8 (11.9)	-	-	-	-	-	-
**Astigmatism**	65 (97)	91 (60)	12 (28.6)	31 (70.5)	38 (30.9)	44 (28)	6 (9)
**Anisometropia**	10 (14.9)	-	-	-	-	-	1 (1.5)
**Slanting fissure**	50 (89.6)	125 (82)	-	-	78 (63)	-	-
**Amblyopia**	36 (54.5)	29 (19)	Presumptive amblyopia (34.4%)	16 (36.4)	-	4 (3)	15 (22%)
**Lens opacities**	26 (39.4)	20 (13)	-	14 (31.8)	4 (3)	18 (11)	1 (1.5)
**Epicanthus**	46 (68.7)	92 (61)	-	-	75 (61)	132 (84)	-
**Nystagmus**	19 (28.4)	29 (18)	2 (4.8)	1 (2.3)	27 (22)	9 (6)	12 (18)
**Suspected keratoconus**	36 (53.7)	-	-	-	0	-	-
**Strabismus**	14 (21.5)	57 (38)	4 (9.5)	10 (22.7)	31 (25)	56 (36)	23 (33.8)
**Nasolacrimal duct obstruction or Dacryostenosis**	13 (19.4)	46 (30)	1 (2.38)	-	21 (17)	35 (22)	-
**Epiphora**	13 (19.4)	-	-	-	-	-	-
**Ptosis**	12 (17.9)	-	-	-	-	-	-
**Blepharitis**	11 (16.4)	45 (30)	1 (2.38)	12 (27.3)	Blepharoconjunctivitis 20 (16)	Blepharoconjunctivitis 6 (4)	-
**Hirschberg off center**	8 (11.9)	-	-	-	-	-	-
**Brushfield’s nodules**	8 (11.9)	79 (52)	-	12 (27.3)	0 (0)	-	-
**Myopic choroidosis**	7 (10.4)	-	-	-	-	5 (3)	-
**Dacryocystitis**	7 (10.4)	-	-	-	-	-	-
**Peripapillary pigmentary alteration**	3 (4.4)	-	-	-	-	-	-
**Confirmed Keratoconus**	3 (4.4)	-	-	-	0 (0)	-	1 (1.5)
**Conjunctival hyperemia**	2 (2.9)	-	Conjunctivitis 8 (19.05)	-	-	-	-
**Subtemporal peripapillary atrophy**	2 (2.9)	-	-	-	-	-	-
**Persistence of myelin fibers**	2 (2.9)	-	-	-	-	-	-
**Exophthalmos**	1 (1.4)	-	1 (2.38)	-	-	-	-
**Epiblepharon**	1 (1.4)	-	-	-	66 (54)^a^	-	-
**Pterygium**	1 (1.4)	-	-	-	-	-	-
**Heterochromia**	1 (1.4)	-	1 (2.38)	-	-	-	-
**Subpapillary RPE atrophy**	1 (1.4)	-	-	-	Focal RPE hyperplasia 2 (2)	-	-
**Engorged and tortuous retinal vessels**	1 (1.4)	-	-	-	-	1 (0.6)	-
**Hypopigmented macula with myopic growth in the optic nerve**	1 (1.4)	-	-	-	-	-	-
**Retinal Vascular arcades with slight tortuosity**	1 (1.4)	-	-	-	-	-	-
**Enlarged Optic Nerve**	1 (1.4)	-	-	-	-	Tilted disc 1 (0.6)	-

*D* Diopter, *SE* Spherical Equivalent

^a^ Epiblepharon caused by pushing epicanthal fold

## Discussion

Down syndrome (DS) is the most common genetic abnormality worldwide [15], with a prevalence in Colombia of 10 cases per 10,000 live births [16]. Several major studies have described the ocular manifestations of DS, demonstrating variations in the findings between different populations (Table 6). The prevalence of ocular features in Colombian children with DS, including refractive errors, was 100%, higher than previously reported in other studies [3]. Despite this, in Colombia, there are no regulations regarding routine screening of children with DS by an ophthalmologist or other ocular professional, as in some regions of the UK [17].

The distribution of ophthalmological features does not have a consistent pattern throughout all populations (Table 6). Colombian children with DS were characterized mainly by an elevated prevalence of refractive errors, amblyopia, lens opacities, and keratoconus (both suspected and confirmed). Even though several abnormalities that are commonly found in children with DS have no functional significance (like Brushfield’s spots, or epicanthus) [1], the characteristic findings in our cohort have functional and therapeutic relevance.

Refractive errors are the most common ocular findings among children, and several studies have reported that they are higher among young children with DS than in controls [18]. The most common refractive errors in children and adolescents with DS are hyperopia and astigmatism, reaching 81.2% and 94.1%, respectively [19, 20]. Our results align with the literature reports as we found that 98.5% of the participants had at least one eye with a refractive error, being astigmatism and hyperopia the most common. Interestingly, when we solely categorized the refractive errors using the SER, 33.6% of the eyes were emmetropes. However, this classification could mask mixed astigmatism, which was present in 18.7% of the eyes. Therefore, some authors have proposed new classifications for ametropias which could help standardize the report in epidemiological studies. For example, with the classification proposed by Galvis et al. [13], the prevalence of emmetropia in our cohort reduced to 2.2%. This should be considered when comparing epidemiological studies regarding the incidence and prevalence of refractive errors in children with DS and in general.

It has been reported that as children with DS get older, visual acuity significantly worsens compared to controls [20]. A previous study evaluating children and adolescents aged 9 to 16 found a mean visual acuity of 0.33 ± 0.18 logMAR [21]. In that case, all children wore the best refractive correction, and none had clinically significant ocular diseases. In our case, the mean VA was below this value for all refractive errors, with and without the best refractive correction (Tables 2 and 3). However, we must consider that we found a high prevalence of amblyopia, strabismus, cataract, and keratoconus suspect, all of which can alter the BCVA.

Although amblyopia is commonly reported in children with DS, with a prevalence varying from 16.9% to 36.4% [19], we diagnosed it in most children (54.5%). This could be explained by the higher prevalence of strabismus, refractive errors of mixed etiology, and anisometropia in our cohort, which are the main causes of amblyopia in DS [22]. Furthermore, the varied cooperative ability in children with DS implies a challenging setting in the standard care for amblyopia, usually done with spectacles and physical occlusion [19].

There is an increased prevalence of congenital cataracts in children with DS [19]. Nonetheless, the prevalence of lens opacities found in Colombian children with DS (39.4%) is significantly higher compared to other studies (see Table 6). Cataracts in DS usually appear between 12 and 15 years of age [8]. The mean age of the participants in our study, which is older than other reports, could be a key factor in understanding this higher prevalence. Another factor could be the pupil dilation before biomicroscopy, which allowed us a more detailed evaluation of the entire lens, including its periphery.

Few studies report the prevalence of keratoconus and keratoconus suspect in the pediatric population with DS. In our study, both variables had a significant prevalence of 4.4% and 53.7%, respectively. DS is a known risk factor for keratoconus; it is 10 to 300 times more likely for keratoconus to occur in individuals with DS [23]. It is doubtful that children too young could develop keratoconus. However, due to the increased risk, children with DS would benefit from monitoring this feature as they mature.

The remaining ocular manifestations (including the upward slanting of the palpebral fissures, epicanthus, nystagmus, strabismus, nasolacrimal duct obstruction, and blepharitis) had a frequency in accordance with previous reports (Table 6). The lower prevalence of Brushfield’s nodules in our study may be related to the higher incidence of dark irides in the Colombian population, similar to what the Brazilian study reported [8]. Other uncommon manifestations were found in less than 5% of the children. These include peripapillary pigmentary alterations, conjunctival hyperemia, subtemporal peripapillary atrophy, the persistence of myelin fibers, exophthalmos, epiblepharon, pterygium, heterochromia, and enlarged optic nerve head, among others. These features must be understood in the particular clinical context of each patient; no literature supports them as a tendency of the population with DS.

The literature is divided against the predominance of gender concerning ocular manifestations, specifically keratoconus and corneal features. Studies have shown that total corneal refractive powers are significantly higher in females, which leads to steeper corneas and a higher probability of having keratoconus [24]. On the contrary, Kristianslund et al. [25] found that more than six studies have reported a predominance of keratoconus in males. Otherwise, in the same study, two studies did not find differences or associations in the diagnosis of keratoconus as in our study [25]. Considering the number of studies and the discrepancies between them, conducting a systematic review with a meta-analysis that summarizes and statistically analyzes this association between keratoconus and DS is recommended.

Our results showed that the female sex was statistically associated with strabismus. However, Makateb et al. did not find significant differences between genders in the prevalence of any ophthalmological disorder [26]. Paradoxically, females used glasses in a significantly higher proportion than males in our population, and the onset was earlier, suggesting a lower risk of refractive amblyopia. However, amblyopia could be explained by the higher prevalence of strabismus and alterations in the Hirschberg test found in girls. Another possible explanation could be the lack of self-care education and lower adherence to treatments (spectacle use) experienced by children with DS, specifically at this transition age. This is supported by studies of inequities in health care access, adherence, and self-care teaching in these populations [27, 28]. However, we cannot confirm this because there are no studies in our country regarding inequities in the care of children with DS at transitional ages.

### Strengths and limitations

Children and adolescents’ information were collected prospectively and protocolized, this avoids the loss of data found in retrospective studies. Nevertheless, although all the examinations were performed uniformly by an expert specialized in pediatric ophthalmology, there could be subtle manifestations not observed on physical examination, for example, in the retinal periphery in patients with nystagmus due to the difficult children’s collaboration.

Likewise, although “Corporación Síndrome de Down” is a recognized referral center in the capital city of Colombia, receiving children from all the sociodemographic backgrounds and social status, a selection bias may occur due to the non-probabilistic sampling because some children that did not accept to participated could be those with better access to the health system and follow-up by the ophthalmologist, even more considering that most of our children proceeded from low- and intermediate- income families.

Additionally, the absence of an age- and gender-matched control group should be noted as a limitation of the study due to the potential influence of demographic factors on ocular findings. Finally, to prevent misclassification and diagnostic bias, an expert was hired to assess all the children, avoiding the influence of co-authorship.

## Conclusions

In our cohort, children and adolescents with DS had a high prevalence of disregarded ophthalmological manifestations. Some of these manifestations, such as amblyopia, can be irreversible and severely affect the neurodevelopment of DS children. Therefore, ophthalmologists and optometrists should be aware of the visual and ocular affection of children with DS to assess and provide appropriate management. This awareness could improve rehabilitation outcomes in children from a low-income level.

## Data Availability

The data that support the findings of this study are available from the corresponding author, CTG, upon reasonable request.
